# Impact of Physical Activity on Diabetes Symptoms and Balance in Individuals with Type 2 Diabetes and Healthy Adults: A Comparative Study

**DOI:** 10.7759/cureus.78838

**Published:** 2025-02-11

**Authors:** Serpil Mıhçıoğlu, Mehtap Malkoç, İlker Yatar

**Affiliations:** 1 Physiotherapy and Rehabilitation, Eastern Mediterranean University, Famagusta, CYP

**Keywords:** balance, diabetes, diabetes symptoms checklist, physical activity, types 2 diabetes

## Abstract

Introduction: Recent studies emphasize that lifestyle changes, including physical activity programs, are at least as effective as pharmacological agents in preventing diabetes. The aim of this study was to compare the body composition, presence of diabetes symptoms, and balance of individuals diagnosed with type 2 diabetes (T2D) and healthy individuals with the same physical activity level.

Methods: A total of 68 individuals participated in the study, including 34 diabetic patients (17 with moderate physical activity (MPA) and 17 with low physical activity (LPA)) and 34 healthy controls (17 with MPA and 17 with LPA). The demographic characteristics of all groups; exercise habits; blood parameters (HbA1c, glucose, cholesterol, high-density lipoprotein (HDL), low-density lipoprotein (LDL), and triglyceride levels); duration of diabetes; body composition; and how the individual was affected by the symptoms of diabetes and balance were evaluated.

Results: There was a significantly worse difference in the diabetes symptoms checklist (DSC) score, body composition, and balance between T2D patients with LPA and healthy individuals (p < 0.05). However, when T2D patients and healthy individuals with MPA were compared, the same parameters were found to be better (p > 0.05).

Conclusion: When individuals with T2D and healthy individuals with the same level of physical activity are examined, it is observed that individuals with T2D who engage in MPA are less affected by complications, while those with low levels of physical activity are more affected. Therefore, we believe that increasing physical activity habits will make a significant positive contribution to the control of diabetes.

## Introduction

Diabetes is a metabolic disease that interferes with the human body’s ability to process and absorb glucose [1]. According to the Heart Disease and Stroke Statistics 2024 Update, 9.7 million adults have undiagnosed diabetes, 29.3 million have diagnosed diabetes, and 115.9 million have pre-diabetes [2].

The cause of type 2 diabetes (T2D) is complex and is known to be influenced by numerous risk factors. According to the American Diabetes Association (ADA), the main goals for T2D patients are good quality of life, good metabolic control, and avoidance of complications. To achieve these goals, the patient's symptoms should be carefully evaluated for good metabolic control and avoidance of complications [3]. Patient-reported outcomes are important in determining the effectiveness of treatments. Raising the awareness of individuals with diabetes, especially about knowing the effects of the symptoms and how to recognize them, is important for diabetes management. Lifelong behavior change, along with education and support, is needed to enable self-management of patients with T2D [4-5].

Considering the benefits of physical activity for the management of T2D, including glycemic control, decrease in diabetes complications, and increase in quality of life, physical activity should be an important therapeutic strategy for the diabetes care team [6]. Regular physical activity can help maintain metabolic health and improve overall health in both healthy individuals and those with diabetes. Therefore, regular exercise is noted to play a critical role in reducing the risk of diabetes and in managing it effectively [7]. It is known that low levels of physical activity can lead to similar symptoms in both healthy individuals and people with diabetes. These symptoms include weight gain, fatigue, muscle weakness, signs of metabolic syndrome, and a decrease in overall quality of life [8].

The decrease in physical activity levels can lead to similar health problems in both healthy individuals and those with diabetes, emphasizing the negative effects of low physical activity (LPA) on metabolic health. Therefore, the aim of this study was to compare the body composition, balance, and presence of diabetes symptoms of individuals diagnosed with T2D and healthy individuals with the same physical activity level.

## Materials and methods

Study design

**Figure 1:**
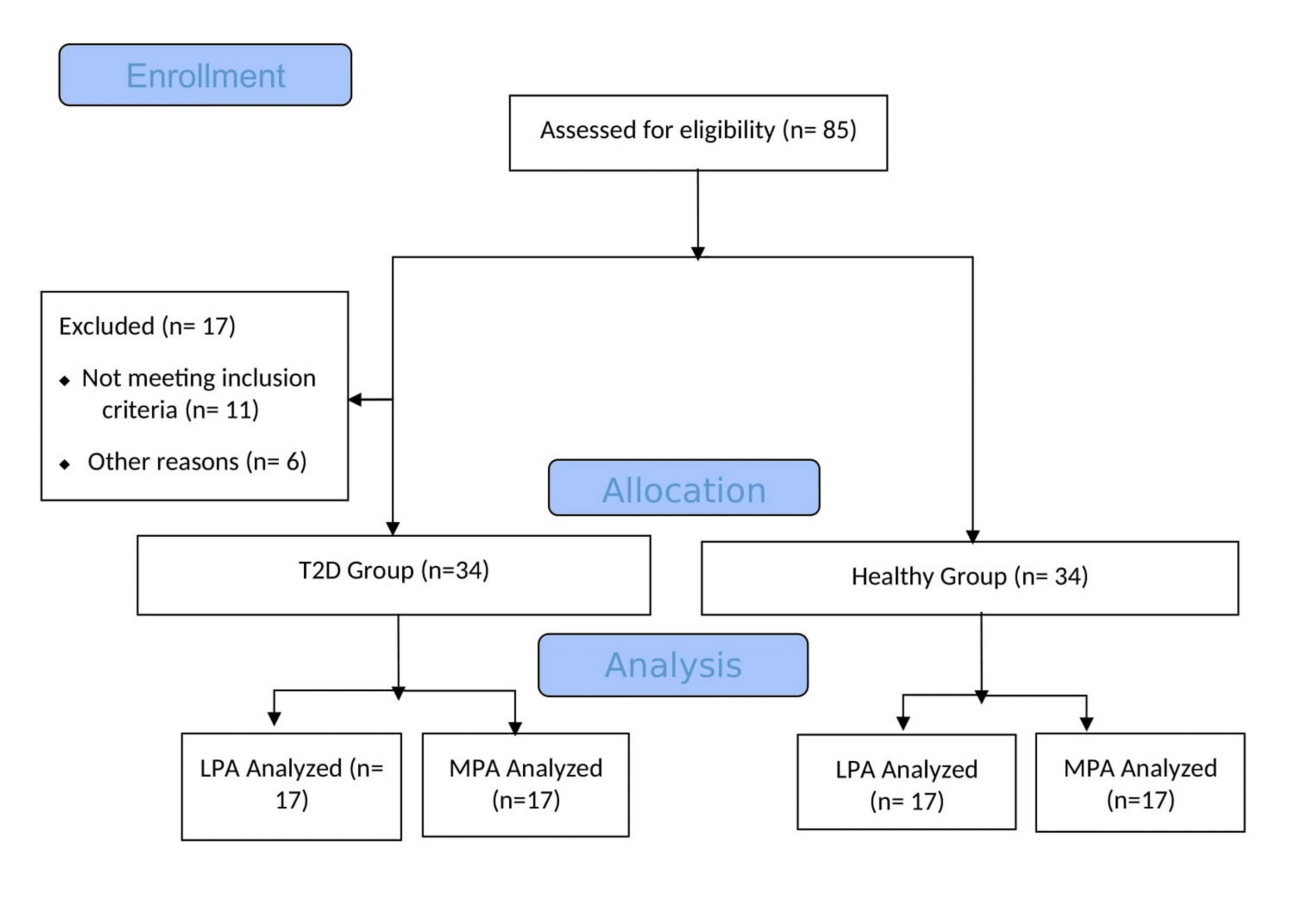
Flow diagram of the study according to CONSORT T2D: type 2 diabetes; MPA: moderate physical activity; LPA: low physical activity; CONSORT: Consolidated Standards of Reporting Trials

This study was performed at the Cardiopulmonary Physiotherapy Unit, Faculty of Health Sciences, Eastern Mediterranean University, Cyprus. This single-blinded, cross-sectional study was conducted between June 2016 and December 2016. The study protocol was approved by the Health Ethics Sub-Committee of Scientific Research and Publication Committee of Eastern Mediterranean University (date: May 15, 2016, no: 2016/27-14). All patients were recruited according to the Declaration of Helsinki; they were informed both orally and in writing, and all gave written consent to participate. The Consolidated Standards of Reporting Trials (CONSORT) flow diagram of the study is shown in Figure 1.

Participants

The sample sizes were 34 diabetic patient groups (17 moderate physical activity (MPA) level, 17 LPA level) and 34 control groups (17 MPA level, 17 LPA level), for a total of 68 individuals (α = 0.05 and β = 0.20, power = 1 - β = 0.80). The physical activity levels of the patients were determined using the International Physical Activity Questionnaire (IPAQ). According to the IPAQ, physical activity should be performed for at least 10 minutes at a time. With the survey in the last seven days, the duration (in minutes) of MPA (carrying light loads, cycling at normal speed, folk dances, dancing, bowling, table tennis, etc.), walking, and sitting times per day (in minutes) were reported by participants.

The total physical activity score (metabolic equivalent of task (MET)-minutes per week (min/week)) was calculated by converting moderate activity and walking times into MET corresponding to the basal metabolic rate with the following calculations:

$\small \text{Walking score (MET-min/week)} = 3.3 \times (\text{walking time}) \times (\text{number of walking days})$ $\small \text {Moderate activity score (MET-min/week)} = 4.0 \times (\text{moderate activity time}) \times (\text{moderate activity day})$ $\small \text {Vigorous activity score (MET-min/week)} = 8.0 \times (\text{vigorous activity duration}) \times (\text{number of vigorous activity days})$ $\small \text {Total physical activity score (MET-min/week)} = \text{walking} + \text{moderate activity} + \text{vigorous activity scores}$

According to the total physical activity score, the participants' physical activity levels were classified as "low or medium." Physical activity levels are categorized as follows: low level, which is less than 600 MET-min/week, and moderate level, which ranges from 600 to 3000 MET-min/week.

Inclusion Criteria

Individuals aged between 40 and 65 years were included in both groups. In addition, those diagnosed with T2D for more than one year, whose glucose control was regulated according to international recommendations, and whose HbA1c level was less than 12% were included in the DM group.

Exclusion Criteria

The exclusion criteria for patients were as follows: congestive heart failure; ischemic and coronary heart disease; cerebrovascular or pulmonary problems; chronic systemic diseases that are contraindications for exercise; hypertension (≥140/90 mmHg or hypertension treatment); severe peripheral or autonomic neuropathy; inability to understand the commands given; and use of medications known to affect heart rate.

Measures

Demographic characteristics of all groups (age, sex, height, weight, BMI, comorbidities (heart and respiratory problems, vision, nutrition, etc.)), family history, and exercise habits (Does he/she exercise weekly?), blood parameters less than three months (HbA1c level of <11%, glucose, cholesterol, high-density lipoprotein (HDL), low-density lipoprotein (LDL), and triglyceride levels), body composition, duration of diabetes, how the individual is affected by the symptoms of diabetes, and balance were evaluated. All assessments were made in a single testing session and were performed by a researcher who was blinded to group allocation.

Body Composition

Waist circumference was measured at the midpoint between the lower margin of the last palpable rib and the top of the iliac crest using a tape measure. For measuring the hips, the position of the greatest circumference at the buttocks was used. The value measured on the tape was recorded in cm for data analysis. For the skinfold thickness measurements, a skinfold caliper (Holtain Ltd., Croswell, England) was used, and the measurements were made on the right side of the triceps, abdomen, and thigh. The value read on the caliper was recorded for data analysis [9].

Diabetes Symptoms Checklist

The diabetes symptoms checklist (DSC) was developed by Grootenhuis et al. (1994) according to the general consensus that quality of life is an important outcome of diabetes care, requiring reliable patient-reported measures pertaining to the physical, social, and psychological domains [10]. The 34 items of the DSC are grouped into six symptom clusters or domains, each measuring a different aspect of diabetes symptomatology: hyperglycemia, hypoglycemia, psychology, cardiology, neurology, and ophthalmology. For each of the 34 DSC items, respondents first indicated whether they had experienced each symptom in the past month. Participants who responded “yes” to the presence of a symptom were subsequently asked to indicate how troublesome the symptom was "not at all, a little, moderate, a lot and extremely." The range of scores that each subparameter can receive is a total of 0-16 points for hyperglycemia (4 items), 0-9 points for hypoglycemia (3 items), 0-24 points for psychological (8 items), 0-16 points for cardiovascular (4 items), and 0-40 points for neurological (10 items) and ophthalmology (5 items) were awarded. In the test, each subtest was scored by taking the average of the items in that section. The total score was determined by taking the average of the scores of the subtests. The calculated subtests and total scores were recorded for data analysis [11].

Balance Assessment

A single-leg standing test was used to assess postural stability. Participants were asked to stand on one foot, barefoot, on a thin sheet, in an upright position facing a wall, and focus their gaze at eye level on a point. They were instructed to lift one leg without touching the supporting leg. Initially, the test was conducted with eyes open, and participants were expected to maintain their balance for 30 seconds. Subsequently, the same procedure was repeated with eyes closed. Loss of balance was considered if the lifted leg touched the supporting leg, the ground, if there was hopping or jumping, or if anything in the surroundings was touched for support [12]. Shortening the duration of standing on one leg indicates reduced balance function, and less than five seconds indicates an increased risk of falls [13]. If the time was longer than 60 seconds, a second measurement was not required. Results were recorded in seconds, and the average of three trials was used for data analysis.

Statistical analysis

The data were analyzed with the IBM SPSS Statistics for Windows, Version 20 (Released 2011; IBM Corp., Armonk, New York, United States). Differences between the means of two independent groups were evaluated using the Mann-Whitney U test, and differences between the means of two dependent groups were evaluated using the Wilcoxon test. The chi-square test and Fisher's exact test were used to evaluate the percentage significance of the difference between groups. Analysis of covariance (ANCOVA) was used to check for covariates. In this study, descriptive statistics for discrete and continuous variables are expressed as the mean ± standard deviation, percent, and number. The statistical significance level was set at p < 0.05. Arithmetic means are given along with 95% confidence intervals (CIs).

## Results

The sociodemographic and clinical characteristics of the participants are given in Table 1. Since there was a difference between the groups in the age data in the study, this difference was used as a covariate, and covariance was controlled with ANCOVA, provided that the prerequisites were met. The duration of diabetes in individuals with T2D, LPA, and MPA levels was not significantly different between the two study groups (p > 0.05). The HbA1c and fasting plasma glucose levels in the diabetes group were significantly greater than those in the healthy group (p < 0.05) (Table 1).

**Table 1:** Comparison of the sociodemographic characteristics of T2D patients and healthy individuals with low and moderate physical activity levels (95% CI) ^*^Mann-Whitney U test; ^†^chi-square test BMI: body mass index; FPG: fasting plasma glucose; HDL: high-density lipoprotein; LDL: low-density lipoprotein; LPA: low physical activity; MPA: moderate physical activity; T2D: type 2 diabetes; x ± sd: mean ± standard deviation

	LPA T2D n = 17	LPA healthy n = 17	p-value	MPA T2D n = 17	MPA healthy n = 17	p-value
Age, year, x ± sd	56.0 ± 8.3 (51.7-60.3)	51.7 ± 7.5 (47.8-55.6)	0.114^*^	56.5 ± 7.3 (52.7-60.3)	50.7 ± 7.1 (47.0-54.4)	0.029^*^
Gender, n (%)						
Female	8 (47.1)	6 (35.3)	0.486^†^	8 (47.1)	7 (41.2)	0.730^†^
Male	9 (52.9)	11 (64.7)		9 (52.9)	10 (58.8)	
Height, m, x ± sd	1.64 ± 0.1 (1.59-1.69)	1.62 ± 0.1 (1.57-1.67)	0.290^*^	1.66 ± 0.1 (1.61-1.71)	1.66 ± 0.1 (1.61-1.71)	0.946^*^
Weight, kg, x ± sd	85.4 ± 12.6 (78.9-1.9)	70.8 ± 13.6 (63.8-77.8)	0.002^*^	79.4 ± 12.9 (72.8-86.0)	76.9 ± 14.2 (69.6-84.2)	0.518^*^
BMİ, kg/m^2^, x ± sd	31.6 ± 3.8 (29.6-33.6)	26.9 ± 4.4 (24.6-29.2)	0.001^*^	29.0 ± 4.3 (26.8-31.2)	27.9 ± 4.3 (25.7-30.1)	0.413^*^
Exercise habits, n (%)						
Yes	7 (41.2)	6 (35.3)	0.724^†^	14 (82.4)	9 (52.9)	0.067^†^
No	10 (58.8)	11 (64.7)		3 (17.6)	8 (47.1)	
Weekly exercise time, min., x ± sd	22.9 ± 32.7 (6.1-39.7)	66.5 ± 128.6 (0.4-132.6)	0.875^*^	124.4 ± 102.5 (71.7-177.1)	117.1 ± 148.6 (40.6-193.5)	0.430^*^
Duration of diabetes, year, x ± sd	7.7 ± 7.9 (3.6-11.8)			7.3 ± 6.5 (3.9-10.7)		0.849
HbA1c%	7.2 ± 1.6 (6.4-8.0)	5.4 ± 0.6 (5.1-5.7)	0.001*	6.9 ± 1.4 (6.2-7.6)	5.2 ± 0.4 (4.9-5.4)	0.001*
FPG, mg/dl	153.1 ± 61.5 (121.5-184.7)	100.8 ± 14.9 (93.1-108.5)	0.001*	160.1 ± 77.2 (120.4-199.8)	97.7 ± 9.9 (92.6-102.8)	0.001*
Total cholesterol, mg/dl	204.1 ± 39.9 (183.6-224.6)	214.9 ± 42.5 (193.1-236.8)	0.394*	233.4 ± 67.5 (198.7-268.1)	212.2 ± 30.5 (196.5-227.9)	0.231*
HDL, mg/dl	44.9 ± 9.6 (39.9–49.8)	51.8 ± 11.7 (45.8–57.8)	0.079*	48.6 ± 14.9 (40.9-56.3)	60.0 ± 36.4 (41.3-78.7)	0.306*
LDL, mg/dl	124.8 ± 40.7 (103.9–145.7)	138.0 ± 33.7 (120.7–155.3)	0.193*	143.2 ± 33.5 (125.9-160.4)	134.6 ± 26.2 (121.1-148.1)	0.586*
Triglyceride, mg/dl	175.0 ± 93.1 (127.1–222.9)	126.2 ± 50.0 (100.5–151.9)	0.062*	164.4 ± 98.9 (113.6-215.2)	135.5 ± 69.1 (99.9-171.0)	0.306*

Body compositions

In individuals with T2D at the LPA level, waist-hip circumference measurements and subcutaneous fat thicknesses at the abdomen and thigh were statistically significantly higher compared to healthy individuals (p < 0.05). When the waist-hip circumference and subcutaneous fat thickness measurements of both groups with MPA levels were compared, there was no difference in waist-hip circumference measurements as well as triceps, abdominal, and thigh skinfold thicknesses compared to healthy individuals (p > 0.05) (Table 2).

**Table 2:** Comparison of circumference and skinfold thickness measurements between T2D patients and healthy individuals with low and moderate physical activity levels, x ± sd (95% CI) ^*^Mann-Whitney U test LPA: low physical activity; MPA: moderate physical activity; T2D: type 2 diabetes; x ± sd: mean ± standard deviation

	LPA T2D n = 17	LPA healthy n = 17	p-value*	MPA T2D n = 17	MPA healthy n = 17	p-value*
Waist circumference, cm	109.1 ± 9.2 (104.4-113.8)	94.8 ± 12.8 (88.2-101.4)	0.001	103.1 ± 10.9 (97.5-108.7)	98.6 ± 11.2 (92.8-104.4)	0.193
Hip circumference, cm	111.1 ± 7.9 (107.3-115.2)	104.4 ± 9.8 (99.4-109.4)	0.034	107.9 ± 8.2 (103.7-112.1)	107.2 ± 9.5 (102.3-112.1)	0.610
Triceps skinfold thickness, mm	21.5 ± 7.8 (17.5-25.5)	18.6 ± 8.0 (14.5-22.7)	0.306	18.7 ± 5.9 (15.7-21.7)	18.6 ± 5.0 (16.0-21.2)	0.812
Abdominal skinfold thickness, mm	27.3 ± 6.9 (23.8-30.8)	21.9 ± 5.1 (19.3-24.5)	0.020	23.2 ± 7.1 (19.5-26.9)	23.4 ± 5.6 (20.5-26.3)	0.973
Thigh skinfold thickness, mm	27.8 ± 9.4 (22.9-32.6)	21.4 ± 8.6 (16.9-25.8)	0.034	21.9 ± 8.9 (17.3-26.5)	26.3 ± 8.5 (21.9-30.7)	0.140

Diabetes symptoms checklist

When the DSC results of T2D patients with LPA levels were compared with those of healthy individuals, statistically significant differences were detected in the hyperglycemia, neurology, cardiology, and ophthalmology subscales and total scores (p < 0.05). When T2D patients and healthy individuals with MPA levels were compared, their scores on the hypoglycemia, neurological, psychological, cardiology, and ophthalmology subscales, and total scores, except for hyperglycemia, showed no significant difference from those of the control group (p > 0.05) (Table 3).

**Table 3:** Comparison of diabetes symptoms checklist scores between T2D patients and healthy individuals with low and moderate physical activity levels, x ± sd (95% CI) ^*^Mann-Whitney U test LPA: low physical activity; MPA: moderate physical activity; T2D: type 2 diabetes; x ± sd: mean ± standard deviation

Scale	Total items	Range of scores	LPA T2D n = 17	LPA healthy n = 17	p-value*	MPA T2D n = 17	MPA healthy n = 17	p-value*
Hyperglycemia	4	(0-16)	5.0 ±3.9 (2.9-7.0)	1.3 ± 2.3 (0.1-2.5)	0.002	3.9 ± 3.7 (1.9-5.8)	1.5 ± 2.9 (0-2.9)	0.012
Hypoglycemia	3	(0-9)	2.2 ± 2.1 (1.1-3.3)	1.6 ± 1.4 (0.9-2.3)	0.413	1.6 ± 1.8 (0.7-2.5)	1.2 ± 1.7 (0.3-2.1)	0.394
Neurology	10	(0-40)	5.0 ± 6.3 (1.8-8.2)	1.9 ± 3.6 (0.1-3.8)	0.034	5.3 ± 5.5 (2.5-8.1)	2.7 ± 3.0 (1.1-4.2)	0.205
Psychology	8	(0-24)	5.9 ± 3.9 (3.9-7.9)	5.2 ± 5.2 (2.5-7.9)	0.375	4.4 ± 3.8 (2.4-6.4)	4.8 ± 5.5 (1.9-7.6)	0.865
Cardiovascular	4	(0-16)	2.6 ± 2.5 (1.3-3.9)	1.0 ± 1.1 (0.4-1.6)	0.049	1.7 ± 2.6 (0.4-3.0)	1.6 ± 2.3 (0.4-2.8)	0.946
Ophthalmology	5	(0-20)	2.3 ± 2.5 (1.0-3.6)	0.7 ± 1.4 (0-1.4)	0.026	2.2 ± 2.3 (1.0-3.4)	1.8 ± 2.9 (0.3-3.1)	0.231
Total	34	Average of the scores	3.8 ± 2.8 (2.4-5.2)	1.9 ± 1.8 (0.9-2.8)	0.041	3.1 ± 2.4 (1.9-4.3)	2.3 ± 2.2 (1.2-3.4)	0.322

Balance

When the eyes open and eyes closed balance times of T2D individuals and healthy individuals with LPA levels are compared, the left side balance times with eyes open are significantly different (p < 0.05), while there is no difference in the right side balance times (p > 0.05). When the eye open-closed balance times of T2D individuals and healthy individuals with MPA levels are compared, there is no difference between eyes open and closed on both sides (p > 0.05). T2D individuals with MPA and healthy individuals with MPA have better eye open-closed balance times (Table 4).

**Table 4:** Comparison of balance test results between T2D patients and healthy individuals with low and moderate physical activity levels, x ± sd (95% CI) *Mann-Whitney U test LPA: low physical activity; MPA: moderate physical activity; T2D: type 2 diabetes; x ± sd: mean ± standard deviation

		LPA T2D n = 17	LPA healthy n = 17	p-value*	MPA T2D n = 17	MPA healthy n = 17	p-value*
Eyes open, time/sec	Left	21.6 ± 20.6 (11.0-32.2)	40.8 ± 28.5 (26.1-55.5)	0.014	35.1 ± 21.7 (23.9-46.3)	59.7 ± 53.3 (32.3-87.1)	0.231
	Right	25.1 ± 20.4 (11.0-31.9)	41.2 ± 30.2 (25.7-56.7)	0.160	37.9 ± 22.1 (26.5-49.3)	63.8 ± 66.7 (29.5-98.1)	0.433
Eyes close, time/sec	Left	3.9 ± 4.4 (1.6-6.2)	5.5 ± 4.4 (3.2-7.8)	0.046	6.5 ± 8.4 (2.2-10.8)	7.8 ± 5.5 (4.9-10.6)	0.085
	Right	3.6 ± 2.6 (2.5-4.7)	5.6 ± 4.9 (3.1-8.1)	0.290	6.8 ± 9.4 (1.9-11.6)	8.9 ± 8.3 (4.6-13.2)	0.454

## Discussion

This study compared body composition, diabetes symptoms, and balance between patients with DM and healthy individuals with the same level of physical activity. Significant differences between DM patients and healthy individuals are particularly notable at low levels of physical activity, and these differences are more pronounced than those at moderate levels. In this study, it was observed that the values of body composition, diabetes symptoms, and balance were worse in individuals with T2D when their physical activity levels were low, while at MPA levels, they were similar to their healthy peers.

Diabetic patients are found to have higher amounts of visceral, subcutaneous, total, and intermuscular adipose tissue compared to healthy individuals [14]. Abdominal obesity is a stronger risk factor for T2D than general obesity. Waist circumference measurements are important in assessing abdominal obesity and are closely associated with T2D and cardiometabolic diseases. The most significant source of chemicals that cause insulin resistance is adipose tissue. Abdominal fat accumulation increases insulin resistance, and lipotoxicity with dysregulated adipokine secretion enhances insulin sensitivity [15,16]. The increase in skinfold measurements, particularly in the abdominal and thigh regions, supports the view that insulin resistance primarily develops through impaired glucose transport in these areas [17]. A study conducted in 2022 evaluated the effects of a sedentary lifestyle on body composition in individuals with T2D. The study found that a sedentary lifestyle could lead to an increase in body fat percentage and trunk fat while causing a decrease in skeletal muscle mass and bone mineral density [18]. Choi et. al. found that trunk and arm fat were higher in individuals with T2D compared to those without diabetes and that each 1 kg increase in body fat raised the risk of diabetes by 15% in men and 19% in women [19]. In our study, when individuals with T2D at LPA levels were compared with healthy individuals, it was observed that the waist and hip circumferences increased in diabetic individuals and fat accumulation in the abdominal and thigh regions was elevated. When individuals with T2D at MPA levels were compared with healthy individuals with the same physical activity level, no significant difference in body composition was observed.

Lifestyle intervention programs for T2D have been validated in numerous controlled studies. It has been determined that physical activity, compared to other treatments alone, has better effects on glycemic control and blood pressure [20,21]. Overall, exercise has a strong beneficial effect on disease symptoms and quality of life in T2D [22-24]. It has been found that individuals who engage in regular moderate-intensity physical activity have a 30% lower risk of T2D compared to sedentary individuals. LPA levels have been associated with poor mental health, depression, insulin resistance, and cardiovascular complications [25-27]. Similar to studies in the literature, in this study, when individuals with T2D at LPA levels were compared with healthy individuals, a statistically significant increase in hyperglycemia, neurological, cardiovascular, and ophthalmological scores was observed in individuals with T2D. When individuals with T2D at MPA levels were compared with healthy individuals, a difference was only found in hyperglycemia symptoms.

Various studies have shown that individuals who develop peripheral neuropathy have difficulty standing on one leg as a result of sensory impairment and decreased distal lower extremity muscle strength [28-29]. Cimbiz and Cakir observed that the one-leg balance test time was lower in individuals with diabetic neuropathy than in healthy individuals [30]. In our study, when the balance results of individuals with T2D with LPA levels were compared with those of healthy individuals, it was shown that the balance times on the left foot with eyes open and closed decreased significantly compared to those of healthy individuals. When the balance results of T2D patients and healthy individuals with MPA levels were compared, the balance times with eyes open and closed and on the right and left feet were found to be similar in both groups. As a result, it can once again emphasize the importance of physical activity, apart from the factors affecting balance.

The limitation of this study is that determining the impact of different treatment strategies (diet, blood glucose-lowering drugs, and insulin) on DSC among diabetes patients was beyond the scope of this study.

## Conclusions

This result shows that LPA levels have more negative effects on individuals with T2D. Body composition, diabetes symptoms, and balance, which are important health indicators, are worse in individuals with T2D compared to healthy individuals at LPA levels. However, when they engage in MPA, these negative effects decrease, indicating that regular physical activity has a beneficial impact on health. This highlights the importance of physical activity for improving the health of individuals with T2D and demonstrates that managing LPA levels is critical in controlling diabetes. Therefore, future studies should carefully examine the effects of interventions aimed at increasing physical activity on symptom distress as a health-related quality-of-life measure and should be supported by long-term follow-up studies.
